# Hsa-circRNA-G004213 promotes cisplatin sensitivity by regulating miR-513b-5p/PRPF39 in liver cancer

**DOI:** 10.3892/mmr.2021.12359

**Published:** 2021-08-11

**Authors:** Ling Qin, Zibo Zhan, Chunxue Wei, Xuemei Li, Tongqin Zhang, Jun Li

Mol Med Rep 23: Article no. 421, 2021; DOI: 10.3892/mmr.2021.12060

Following the publication of the above article, the authors have realized that, on p. 8, some of the supplementary data were cited incorrectly in the main text. In the right-hand column, second paragraph, the sentence beginning on line 5 should have read as follows (changed text is highlighted in bold): “Meanwhile, the expression of miR-513b-5p in tumor tissues was decreased and the expression of PRPF39 was increased in tumor tissues with knockout of circ-G004213 (**Fig. S3D and E**).” (i.e., the reference to Fig. S3C and D was incorrect.)

The authors regret their oversight in failing to correct the inaccurate citation of the data in the paper, are grateful to the Editor for allowing them the opportunity to publish this Corrigendum, and apologize to the readership for any inconvenience caused.

